# Bioactive DNA from extracellular vesicles and particles

**DOI:** 10.1038/s41419-020-02803-4

**Published:** 2020-07-27

**Authors:** Ethan Z. Malkin, Scott V. Bratman

**Affiliations:** 1 Department of Medical Biophysics, University of Toronto, Toronto, ON Canada; 2 Department of Radiation Oncology, University of Toronto, Toronto, ON Canada; 3 Princess Margaret Cancer Centre, Toronto, ON Canada

**Keywords:** Organelles, DNA metabolism, Inflammation

## Abstract

Extracellular vesicles (EVs) and particles (EPs) have recently emerged as active carriers of molecular biomarkers and mediators of intercellular communication. While most investigations have focused exclusively on the protein, lipid and RNA constituents of these extracellular entities, EV/EP DNA remains poorly understood, despite DNA being found in association with virtually all EV/EP populations. The functional potential of EV/EP DNA has been proposed in a number of pathological states, including malignancies and autoimmune diseases. Moreover, the effectiveness of cell-free DNA as the biomarker of choice in emerging liquid biopsy applications highlights the role that EV/EP DNA may play as a novel disease biomarker. In this review, we provide a comprehensive overview of EV/EP DNA studies conducted to date, with a particular focus on the roles of EV/EP DNA as a functional mediator and molecular biomarker in various pathologic states. We also review what is currently known about the origins, structure, localisation and distribution of EV/EP DNA, highlighting current controversies as well as opportunities for future investigation.

## Facts

- Distribution of genomic and mitochondrial DNA differs between unique subpopulations of EVs and EPs
- EV/EP DNA varies in its structure (i.e. fragment length, conformation and binding proteins) and localisation (i.e. membrane-bound, membrane-associated and protein-bound)
- EV/EP DNA is involved in pathological intercellular communication in cancer and immune diseases, among others
- As a type of cell-free DNA, EV/EP DNA is amenable to liquid biopsy applications for disease detection and monitoring

## Open questions

- What intracellular processes are involved in packaging DNA into different EV/EP subpopulations?
- Do DNA structure, intracellular origin (i.e. nuclear vs. mitochondrial) and localisation influence its function in recipient cells?
- Are the phenotypic changes induced by EV/EP DNA in pre-clinical models also seen in clinical models?
- Does EV/EP DNA present any advantages over cell-free DNA as a source of molecular biomarkers in liquid biopsy approaches?
- Of the many EV isolation and analysis techniques currently in use, which are most appropriate for studies of EV/EP DNA?

## Introduction

Cells are constantly releasing large quantities of molecular material, much of which acts directly or indirectly in intercellular signalling. These extracellular components can modulate physiological processes and be noninvasively accessed from biofluids to serve as molecular biomarkers. In particular, extracellular cell-free DNA (cfDNA) has seen a meteoritic rise in research interest and clinical applications because of its demonstrated potential as an effective biomarker in cancer and other diseases^1–3^. In addition, extracellular DNA has been implicated in physiological processes in various diseases. For example, neutrophils release DNA-rich chromatin webs termed neutrophil extracellular traps (NETs), which can drive disease progression in cancer, systemic lupus erythematosus (SLE), and thromboembolic conditions^4–7^. Other forms of cfDNA, such as nucleosomes, interact with various cells and extracellular components to influence cellular processes related to tumourigenesis and metastasis^1,8^. Moreover, cfDNA can be genomic (gDNA) or mitochondrial (mtDNA) in origin, and this distinction can influence functionality. These unique DNA species differ in their bound proteins, nucleic acid structure, and genetic material, with implications for both their physiological functions and use as molecular biomarkers^9–12^.

Extracellular DNA release can occur through apoptotic and necrotic cell death^13^. Subsequent evidence has supported active secretion/extrusion of DNA in the form of nucleosomes and other nucleic acid-protein complexes^1,14^, including NETs^15^. Despite longstanding knowledge of the existence of extracellular vesicles (EVs) as a mode of active release of cellular contents, evidence suggesting that EVs contained DNA only emerged within the past decade. Initial studies focused on double-stranded DNA within EVs from cell line conditioned media^16,17^. Since then, EV DNA has quickly become a widely studied novel molecular biomarker and functional mediator in various physiologic and pathologic states. This explosion in EV DNA-related research has created a still nascent field lacking validated protocols, standardised nomenclature, and consistent findings^18,19^.

Here, we aim to present a comprehensive review of the role of EV DNA as a functional mediator and molecular biomarker. Specifically, we review the nomenclature of EVs and extracellular particles (EPs), the physical and structural characteristics of EV/EP DNA, the physiological roles of EV/EP DNA in health and disease and the emerging potential of EV/EP DNA as a molecular biomarker.

## Extracellular vesicles and particles

Virtually all human cells release EVs, lipid-bilayer-encapsulated parcels that carry a diversity of molecular cargo. EVs comprise a heterogenous population, with differences in biogenesis, size and contents between different subpopulations. Based on these parameters, EVs are generally classified as either large (large extracellular vesicles (L-EVs)), including apoptotic bodies, large oncosomes and microvesicles; or small (small Extracellular Vesicles (S-EVs)), including exosomes. However, these designations often oversimplify the diversity of EV populations and have created confusion in the field of EV research^20,21^.

Several isolation methods are used to distinguish between EV populations. Differential ultracentrifugation separates L-EVs and S-EVs based on their densities; S-EVs can then be further separated into subpopulations by spinning on a sucrose density gradient. Other widely used methods, such as size exclusion chromatography and ultrafiltration, allow for enrichment of EVs of specific sizes and molecular weights, respectively^18,22^. These established methods have recently been supplemented with newer technologies such as nanoscale flow cytometry and microfluidics, which sort EVs based on their surface markers and size^18,23–25^. The lack of standardisation in EV isolation techniques has made evaluating and comparing findings difficult^20^.

**Fig. 1:**
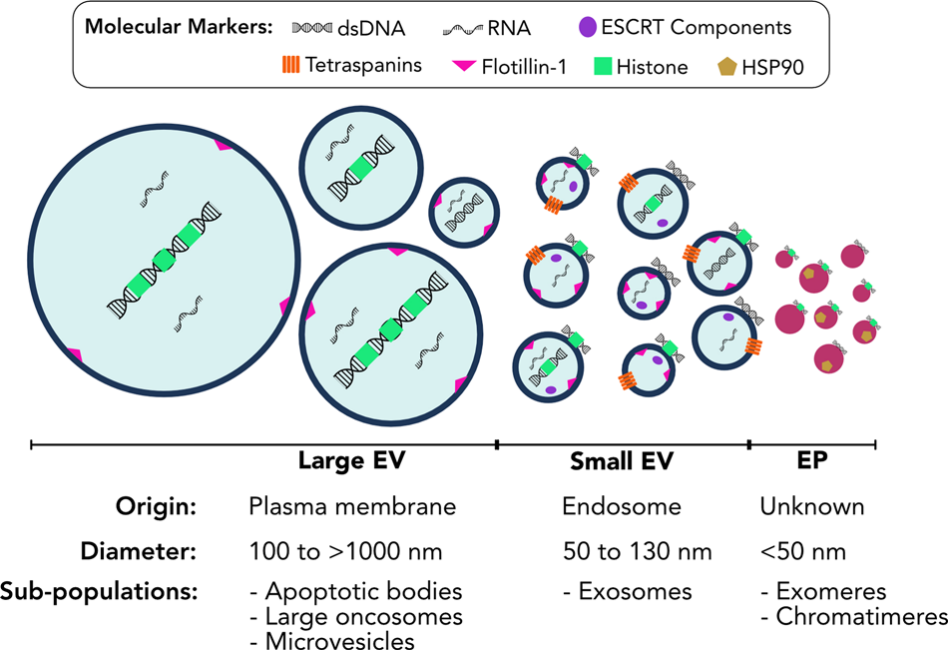
Extracellular vesicles and particles comprise heterogenous populations. Large extracellular vesicles (L-EVs) are characterised by their formation from the plasma membrane, their predominantly large size, and the long fragment length of their luminal DNA. Small extracellular vesicles (S-EVs) are primarily characterised by their endosomal origins, surface protein markers, and smaller size, though overlap in size does exist between larger S-EVs and smaller L-EVs. Extracellular particles (EPs) are small protein-nucleic-acid complexes that are not membrane-bound; the mechanisms of EP biogenesis are unknown. Within each of these groups, multiple subpopulations have been described. However, the full extent of EV/EP subpopulation heterogeneity has yet to be realised, as exemplified by the differences in size and molecular markers both between and within different EV/EP populations. *ESCRT* endosomal sorting complexes required for transport, *HSP90* heat shock protein 90.

EPs represent another group of cell-secreted entities that lack membranes but may have overlapping structure and/or function with EVs, adding yet another layer of complexity to extracellular contents. Importantly, these heterogenous populations not only differ in their origins and biophysical characteristics, but also their DNA content and functional properties. As such, a brief overview of EV/EPs is required to fully grasp the diverse roles of EV/EP DNA to be discussed (Fig. 1).

### Large extracellular vesicles

L-EVs are characterised primarily by their size (>200 nm diameter) and their formation from the plasma membrane. L-EV biogenesis is largely regulated by plasma membrane-associated cytoskeletal proteins, cytosolic loading complexes, and membrane lipids^26,27^. Apoptotic bodies are the largest cell-secreted vesicles (>1000 nm) and are released during apoptosis^28^. Because they are not actively released from live cells, apoptotic bodies are not usually classified as EVs, though they are widely studied alongside the more classical EV populations. Large oncosomes are similar in size to apoptotic bodies but consist of a distinct proteomic profile^29^. As the name implies, large oncosomes have primarily been described in the context of cancer; it remains unclear whether they are actively released by cells or form as a by-product of cell death^18,29^. Microvesicles range from <100 nm to >1000 nm in diameter, making them the most diverse EV population in terms of size^30^. Despite their broad range in size, microvesicles nonetheless form from budding of the plasma membrane, and will therefore be classified as L-EVs for the sake of simplicity and consistency. While these L-EV populations share similar sizes and general mechanisms of biogenesis, their molecular cargos (including DNA content, as described in Section 3.2) and functional roles vary^18,30^.

### Small extracellular vesicles

S-EVs are usually smaller (50–130 nm) than L-EVs but with some overlap. S-EVs are therefore more appropriately distinguished by their mechanism of biogenesis. S-EV formation begins at the surface of the late endosome, where the endosomal membrane invaginates and cytoplasmic contents are loaded into newly formed intraluminal vesicles^31^. These vesicles are released into the extracellular space as S-EVs upon fusion of the late endosome (at this point termed a multivesicular body) with the plasma membrane. While the endosomal mechanism of S-EV formation is widely accepted, some evidence suggests that S-EVs can also form directly from the plasma membrane, or from intracellular compartments connected via a long channel to the cell surface^32^. The complexity of S-EV biogenesis enables regulation at multiple steps: vesicle loading, controlled by endosomal sorting complex required for transport (ESCRT) pathway proteins; intracellular transport, mediated by cytoskeletal complexes and cytosolic GTPases; and release from the plasma membrane, regulated by membrane-associated protein receptor complexes^26,27,31^. These regulatory factors may influence functional differences between S-EVs and other vesicles and particles (Tables 1–3).

**Table 1:** EV/EP DNA distribution by study.

Disease model	EV/EP source	EV/EP	DNA origin	Refs
n/a (non-mammalian)	Cell culture	L-EV	Unspecified	^55^
Various cancers	Cell culture, mouse plasma	L-EV	Genomic	^59^
Systemic lupus erythematosus	Cell culture	L-EV	Genomic	^46^
Prostate cancer	Cell culture, human plasma	L-EV, S-EV	Genomic	^58^
Various cancers	Cell culture	L-EV, S-EV	Genomic	^60^
Healthy	Human plasma	S-EV	Genomic	^56^
Bacterial infection	Cell culture	S-EV	Genomic, mitochondrial	^52^
Prostate cancer	Cell culture, human plasma	L-EV	Genomic	^57^
Various cancers	Cell culture	S-EV, EP	Unspecified	^34^
Various cancers	Cell culture	S-EV, EP	Genomic	^41^
Various cancers	Cell culture	EP	Unspecified	^61^
Leukaemia	Cell culture	S-EV	Genomic, mitochondrial	^35^
Various cancers	Cell Culture	S-EV, EP	Unspecified	^40^

**Table 2:** EV/EP DNA function by study.

Disease model	EV/EP DNA source	Functional mechanism	Molecular changes	Outcomes	Refs
Chronic myeloid leukaemia	Tumour cell	HGT of gDNA	↑ AT-1 expression	↑ Oncogenesis	^93^
Coronary artery disease	Plasma	SRY HGT to monocytes and endothelial cells	↑ CD11a, THP-1, iCAM expression	↑ Cell adhesion, atherosclerosis	^94^
N/A	Bone marrow mesenchymal cell	HGT of gDNA	Exogenous DNA expression	N/A	^60^
Cancer chemotherapy	Enterocyte	Macrophage DNA receptor activation	AIM2 inflammasome activation	Cytokine release, enterocyte inflammation	^90^
Breast cancer	Cancer-associated fibroblast	HGT of mtDNA	↑ Oxidative phosphorylation	Escape from metabolic quiescence	^95^
Malaria	Infected erythrocyte	Monocyte DNA receptor activation	cGAS/STING activation	Innate immune response	^85^
Cancer radiotherapy	Tumour cell	DC DNA receptor activation	cGAS/STING activation	CD8 + T cell priming	^81^
Anti-viral immunity	Activated CD8 + T cell	DC DNA receptor activation	cGAS/STING activation	DC anti-viral priming	^66^
Autism spectrum disorder	Plasma	Unspecified	↑ IL-1β release	Microglia inflammation	^89^
Cancer radiotherapy	Fibroblast	Radiation-induced bystander effect	mtDNA damage	gDNA damage of recipient cell	^92^
Breast cancer	Serum	HGT of HPV DNA	↑ CD44, IL-6 release	↑ Tumour aggression	^96^
Liver cancer	Tumour cell	Oncogenic pathway activation	↑ JNK, STAT3, BCL-2, TAZ signalling	↑ Oncogenesis	^91^

**Table 3:** EV/EP DNA as a biomarker by study.

Disease	EV/EP source	Mutations detected	Refs
Renal disease	Urine	N/A	^114^
Pancreatic cancer	Serum	*KRAS, P53*	^17^
Melanoma	Plasma	*EGFR, BRAF*	^16^
Pancreatic cancer	Serum	*KRAS*	^103^
Glioma	Peripheral blood	Multiple	^109^
Pancreatic cancer	Serum	*KRAS, P53*	^104^
Pancreatic cancer	Serum	*KRAS*, others	^105^
NSCLC	Bronchioalveolar lavage fluid	*EGFR*	^113^
Bladder cancer	Urine	Multiple	^115^
NSCLC	Malignant pleural effusion	*EGFR*	^110^
Multiple advanced cancers	Plasma	*BRAF, EGFR, KRAS*	^100^
NSCLC	Malignant pleural effusion	*EGFR*	^101^
Pancreatic cancer	Serum	*KRAS*	^106^
Ovarian cancer	Plasma	mtDNA copy number	^102^
NSCLC	Malignant pleural effusion	*EGFR*	^111^
NSCLC	Malignant pleural effusion	Multiple	^112^
Pancreatic cancer	Plasma	*KRAS*	^107^

*NSCLC* non-small-cell lung cancer.

Until recently, S-EVs were almost exclusively referred to as exosomes, classically defined as a subset of EVs with an average size of 100 nm and expressing the surface markers CD9, CD63, and CD81^32,33^. However, the term “exosome” casts far too broad a net when describing S-EVs^21^. Unique S-EV subpopulations have been described in terms of their size^34^, density^35,36^, and protein composition^37,38^, challenging the notion that exosomes exist as a single homogenous population. This lack of clarity surrounding S-EV heterogeneity can be at least partially attributed to the plethora of different isolation and characterisation techniques used to separate and describe these vesicles^18–20,22^. Moreover, the small size of S-EVs itself presents a challenge for some existing methods to distinguish and characterise these entities^18,19,22,39^. Until high-resolution separation of S-EVs can be optimised and approaches to S-EV analysis standardised, our understanding of S-EV heterogeneity will remain murky (Boxes 1 and 2).

Box 1 DNA: the overlooked component of EV/EPs Despite a general acceptance of DNA as a constituent of EVs and EPs, its utility and potential importance is often overlooked. Multiple EV component databases, which compile specific protein, lipid and RNA markers identified in EV studies, notably omit DNA. Interestingly, RNA has been extensively studied as a potential EV-based molecular biomarker despite DNA being the predominant nucleic acid marker in many liquid biopsy approaches. Perhaps EV/EP DNA studies lag behind their protein, lipid and RNA counterparts because DNA was first described only a few years ago, in two different studies by Thakur et al. and Kahlert et al. in 2014. Since its discovery, EV/EP DNA has been implicated in various disease processes both as a mediator of physiological function and a potential biomarker in liquid biopsy applications. However, the lack of foundational research on EV/EP DNA biophysical properties and subpopulation localisation has likely delayed the emergence of EV/EP DNA into the spotlight. As the EV field continues to gain momentum, a full understanding of EV/EP DNA will be crucial in broadening and solidifying our knowledge of EV/EPs on the whole.

Box 2 EV/EP DNA unanswered questions While a general understanding of EV/EP DNA is beginning to emerge, several important questions regarding biogenesis, structure, localisation and function, remain unanswered. Here, we highlight some of these questions that must be addressed if EV/EP DNA is to be used successfully as a clinical biomarker and potential therapeutic target:

- What intracellular processes are involved in packaging DNA into different EV/EP populations and subpopulations? How do these loading pathways differ with regards to processing genomic versus mitochondrial DNA?
- How is DNA structurally associated with EV/EPs? Is the DNA found within the EV lumen or on the outer membrane? If DNA is associated with the outer membrane, what is mediating this interaction?
- Does DNA structure (i.e. fragment length, higher-order structure) differ between EV/EP populations? How do structural differences relate to biogenesis and loading? Does DNA structure influence its function in recipient cells?
- To which EV/EP populations does DNA localise? Does EV/EP DNA localisation differ by cell type of origin or pathological state?
- With what pathways does EV/EP DNA interact in recipient cells? Do the phenotypic changes resulting from pathway activation translate to clinical models?
- How much of cfDNA is associated with EV/EPs? What are the implications of this cfDNA localisation on liquid biopsy applications?
- Which isolation and analysis techniques are most amenable to EV/EP DNA studies?

### Extracellular particles

EPs are a relatively novel concept in the field of EVs, as advances in isolation and characterisation technologies have only recently allowed for EP identification. One such technology, asymmetric-flow field-flow fractionation, was employed in isolating a population of EPs termed “exomeres”, with a mean diameter of 35 nm and conserved molecular composition across different cell types^34^. EPs have also been observed using more conventional methods^40^. A second unique EP population referred to as “chromatimeres” was recently described using a nanoscale flow cytometry approach; however, the true nature of chromatimeres as non-membrane-bound particles remains to be confirmed^41^. Due to the nascency of these discoveries, little is known about EP biogenesis, structural and biological composition, mechanism of release, and functionality. Moreover, it remains unclear whether the term “extracellular particle” encompasses all cell-secreted nucleic acid-protein complexes; if this is the case, then previously described complexes such as nucleosomes and mitochondrial nucleoids should be considered EPs as well. These questions must be addressed to distinguish these newly described structures from other components of the cellular secretome.

Despite the heterogenous nature of EV/EPs, every population described to date has been shown to associate with DNA. Therefore, EV/EP DNA represents a key component of these extracellular entities with potential roles in EV/EP-mediated function and liquid biopsy strategies.

## Biophysical properties of EV/EP DNA

**Fig. 2:**
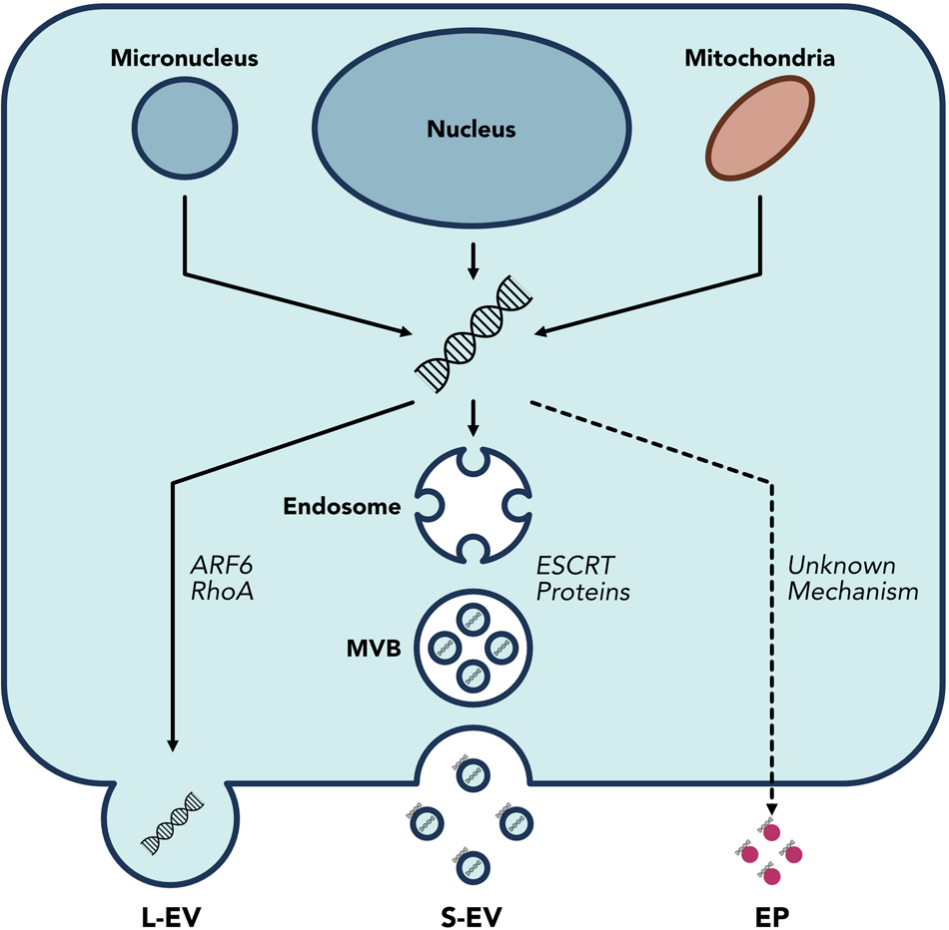
DNA loading mechanisms are unique for different EV/EP populations. DNA is released into the cytosol from the nucleus and micronuclei in response to genomic instability and DNA damage; mitochondria can also release DNA into the cytosol. The precise mechanisms by which DNA is loaded into EVs are unknown, but ARF6 and RhoA have been implicated in L-EV DNA loading, while the ESCRT family of proteins are involved in S-EV DNA loading. The mechanisms of DNA loading onto EPs remains unknown. Moreover, the means by which DNA associates with the surface membrane of S-EVs is not fully understood. Consequently, EV/EP DNA loading mechanisms and structure represent areas that require further study.

Despite the intensive study of EV/EPs as potential sources of disease biomarkers and mediators of physiological function, the associated DNA remains largely unexplored. Studies of EV/EP structure and function have to this point focused almost exclusively on their protein, lipid and RNA cargo and composition^32,42,43^. This oversight is aptly highlighted by two recently published databases that document hundreds of proteins, lipids and RNA species proposed as exosome components in various studies, with the stark omission of DNA^44,45^. Although it is widely accepted that EV/EPs carry DNA cargo, the origins, localisation and biophysical properties of this DNA are not entirely understood. In this section, we explore what is known about the biophysical and structural properties of EV/EP DNA (Fig. 2).

### Origins and loading

L-EVs originate at the plasma membrane, where outward vesicle formation allows for the encapsulation of cytoplasmic contents. This seemingly non-specific loading strategy is mostly applicable to apoptotic bodies, which were shown to contain DNA reflective of their cell of origin in an early study on L-EV biogenesis^46^. Meanwhile, biogenesis of other L-EV populations is mediated by ADP-ribolsylation factor 6 (ARF6) and Ras homologue family member A (RhoA), though the link between these loading mechanisms and L-EV DNA remains unclear^18^.

Meanwhile, S-EVs employ more complex loading mechanisms controlled by ESCRT pathway proteins. Thus far, DNA has not been specifically implicated as a substrate of these loading mechanisms. Speculation surrounding the loading of DNA into S-EVs has focused largely on micronuclei, small buddings of the nucleus resulting from DNA damage response pathways^47,48^. When micronuclei membranes rupture, the DNA within is released into the cytosol. Yokoi et al.^49^ demonstrated co-localisation of tetraspanins (an established EV marker) with micronuclei markers. Moreover, they found that treatments that induced genomic instability caused an increase in micronuclei formation, which correlated with an increase in EV DNA levels. Interestingly, only a small proportion of EVs in their study (10% of cell line-derived EVs and 1% of plasma-derived EVs) actually contained gDNA, suggesting that loading of nuclear contents into EVs may not be efficient or widespread. Nonetheless, these results support those of an earlier study that proposed DNA secretion via EVs as an integral mechanism in clearing cytosolic DNA to maintain cellular homeostasis and avoid senescence and apoptotis^50^.

While micronuclei may play a role in loading gDNA into EVs, the origins of EV/EP mtDNA cannot be explained by this mechanism. In fact, of the few studies that have focused exclusively on EV/EP mtDNA, none directly explore the mechanisms by which mtDNA is packaged and secreted^35,51,52^. In reviewing intra- and extra-cellular mtDNA secretion, Perez-Trevino et al.^53^ theorised that mitochondria-derived vesicles generated from oxidative damage could be targeted to endosomal pathways, where their DNA contents are packaged into S-EVs^53^. Picca et al.^54^ further postulated that lysosomal vesicles resulting from mitochondrial autophagy, and thus rich in mitochondrial contents, may themselves be released as EVs. More work is needed to elucidate the ways in which both gDNA and mtDNA are loaded into and released via EV/EPs.

### Distribution and localisation

Two contentious topics surrounding EV/EP DNA include which populations are actually associated with DNA (i.e. distribution) and whether the DNA is located within the membrane-enclosed vesicular lumen or associated with the outer EV membrane (i.e. localisation). An early study of the primitive eukaryote Dictyostelium discoideum found that L-EVs secreted by this unicellular organism were associated with large fragments of dsDNA (>21 kb)^55^. In the context of SLE, apoptotic bodies were implicated as carriers of dsDNA within the vesicular lumen^46^. However, neither of these studies quantified DNA in S-EVs or EPs. Conversely, S-EVs were suggested to contain up to 93% of plasma cfDNA in a study that also lacked appropriate controls and comparisons^56^.

More recent work has focused on comparing the DNA content of EVs across different populations. Vagner et al.^57^ found that gDNA was primarily found in L-EVs (specifically large oncosomes) derived from prostate cancer plasma and cell culture media. Interestingly, L-EV DNA exhibited fragment lengths of >2 million bp, suggesting the presence of higher-order DNA structure. These L-EVs also contained ssDNA, and both ssDNA and dsDNA were shown to be somewhat protected against degradation. In another comparative study, L-EVs and S-EVs were isolated from the plasma of prostate cancer patients, and the presence of prostate cancer-specific mutations was used as a surrogate for DNA presence^58^. While this study found mutated DNA within both L-EVs and S-EVs, the lack of total gDNA and mtDNA quantification casts uncertainty over the true DNA distribution. This issue is highlighted by an earlier study in which microvesicles were proposed as the primary extracellular carriers of gDNA, despite a lack of DNA characterisation and use of c-Myc mutation copy number as a proxy for total EV DNA^59^. In any case, these studies collectively suggest that L-EVs do contain gDNA in their vesicular lumen, though the relative proportion of cfDNA contained in these vesicles requires further study.

The distribution of DNA within S-EVs also remains contested, although most agree that S-EV DNA is predominantly localised to the outer membrane of S-EVs as opposed to the intraluminal space^35,52,60^. Lazaro-Ibanez et al. isolated high- and low-density S-EVs from mast cell and leukaemic cell cultures using differential gradient centrifugation. While both fractions contained DNA, the high-density S-EVs harboured a greater proportion of both mtDNA and gDNA^35^. The presence of mtDNA and gDNA on the vesicle surface supports earlier findings that S-EVs are associated with both types of DNA^52^, but it also implies differences in DNA distribution among S-EV subpopulations.

EPs have recently been championed as potential carriers of large amounts of cfDNA. In a landmark study, Jeppesen, et al.^61^ used a high-resolution ultracentrifugation and density gradient technique to isolate S-EVs and found that S-EVs did not contain detectable amounts of DNA. Surprisingly, they instead found that the majority of isolated DNA was associated with non-vesicular particles that left the DNA completely unprotected to degradation. These results were in part corroborated shortly thereafter with the discovery of exomeres by Zhang et al.^34^, in which these newly described EPs were shown to carry gDNA. However, their isolation strategy also yielded small and large subpopulations of S-EVs that were both associated with gDNA. The DNA concentration of each EV/EP population differed depending on the cell line of origin, but in every case each entity was associated with at least some DNA. Interestingly, the Coffey group presented data in a subsequent study that supported findings of more widely dispersed DNA content across S-EV and EP subpopulations posited by Zhang et al.^40^, despite having employed the same approach as their original study.

Another recently described DNA-containing EP is the chromatimere. This particle was identified by staining samples with a membrane-impermeable DNA dye and analysing by nanoscale flow cytometry^41^. Proteomic characterisation confirmed a lack of classical S-EV markers and the presence of histones associated with these particles, suggesting a nuclear origin for the DNA. However, further work is needed to clarify whether these particles are actually non-membranous particles, or rather an S-EV subpopulation with surface-associated histone-bound DNA.

Clearly, the distribution of cfDNA in EV/EP populations is complex, and no consensus has been reached as to its true localisation. These somewhat confusing differences in findings may have as much to do with the array of EV/EP isolation and DNA characterisation methods as they do with the heterogeneity of EV/EP DNA itself^18–20,22^. Therefore, a consensus must first be reached regarding EV DNA isolation and characterisation approaches before an understanding of the true nature of extracellular DNA localisation can be achieved.

### Structural and biophysical properties

The biophysical characteristics of EV/EP DNA have broad implications for its use as a molecular biomarker and its capacity to mediate physiological functions. Given its potential importance, surprisingly little is known about the structural aspects of EV/EP DNA. Studies conducted to date have focused primarily on DNA fragment length, DNA-binding proteins and DNA membrane association to elucidate its biophysical properties. As alluded to earlier, EV/EP DNA fragment lengths vary greatly and are largely dependent on the size of the EV/EP with which they are associated. The DNA can range in size from ~200 bp in S-EVs^35,50,56^ to >2 million bp in L-EVs^57^. While these larger fragments have been shown to be chromosomal in nature, many of the smaller EV DNA fragments were also shown to bind histones or other proteins. Interestingly, EP DNA was found to range from 100 bp up to 10 kb, despite EPs being smaller than their EV counterparts^34^. Whether the fragment length discrepancies between unique EV/EP populations imparts functional differences remains to be seen.

Another important aspect of EV DNA structure is its association with the outer vesicular membrane surface. Studies of both bacterial and mammalian EVs have reported DNA as a component of the vesicular “surfaceome”^62^. In bacteria, membrane vesicles (analogous to eukaryotic L-EVs) were found to carry DNA on the vesicular surface^63,64^. However, the mechanisms by which cytosolic DNA reaches the outer cell membrane were not addressed in these studies. In fact, there exists a rather glaring lack of clarity regarding the fundamental question of how DNA associates with the EV lipid bilayer. The current gold standard experiment used to demonstrate the presence of surface EV DNA involves treating purified EVs with DNase I with and without a membrane-disrupting detergent; a significant decrease in DNA concentration after DNase I treatment is interpreted as confirming the presence of unprotected DNA, and thus DNA associated with the vesicular membrane^20,35,52,61^. However, the actual biochemical interaction mediating this association has yet to be described, and very few studies have demonstrated DNA-membrane interactions in any context^65^. Therefore, more work must be done to elucidate whether EV-DNA binding is mediated by membrane proteins, glycoproteins, modified phospholipids, or some other mechanism. Similarly, the biophysical aspects of DNA binding remain a mystery in the newly discovered EP populations.

## EV/EP DNA as a functional mediator

**Fig. 3:**
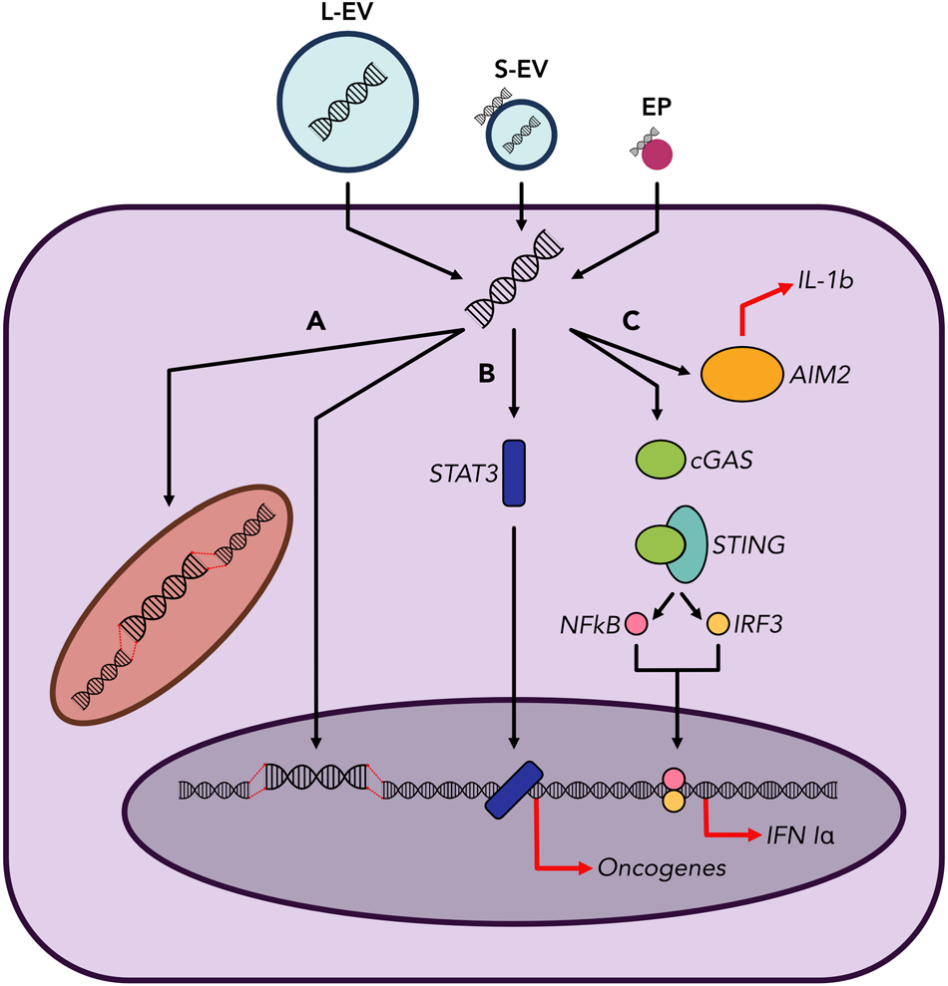
EV/EP DNA directly influences physiological function by acting on recipient cells. EV/EPs deliver DNA to the recipient cell cytosol. **a** Horizontal gene transfer. EV/EP DNA can translocate to the recipient cell nucleus or mitochondria, where it is integrated into the host genome. Subsequent transcription of this DNA heavily influences recipient cell function, and phenotypic changes depend on the genotype of cells from which the EV/EP DNA was derived. **b** Activation of oncogenic pathways. EV/EP DNA can activate or cause the up-regulation of various intracellular signalling proteins, such as STAT3, causing translocation to the nucleus and over-expression of oncogenes that drive a pro-tumourigenic phenotype in the recipient cell. **c** Activation of inflammatory pathways. EV/EP DNA can trigger various cytosolic DNA receptors, including AIM2 (which subsequently produces interleukins) and cGAS. Activation of cGAS causes downstream release of Type I Interferons that induce inflammatory responses unique to the disease context.

Intercellular communication is essential in both normal physiological processes and the development of disease. As such, EV/EPs represent a powerful mechanism by which cells can exchange material and information both locally and at distant sites. EV/EP-mediated intercellular communication has been implicated as an important functional mechanism in immunity, with EV/EP DNA specifically being proposed as a mediator of the innate and adaptive immune systems^66,67^. The physiological role of EV/EP DNA has also been explored in the context of maintaining cellular homeostasis, whereby cells excrete potentially harmful damaged DNA to avoid induction of apoptosis^50^. However, additional roles of EV/EP DNA in normal health remain largely unknown beyond these studies. Moreover, while EV/EPs are also involved in a variety other of physiological processes, including embryogenesis, foetal-maternal crosstalk, and thrombosis, the molecular mechanisms by which EV/EPs interact with and are taken up by recipient cells require further study^42,68,69^. Nonetheless, the contents of these entities have been shown to interact with receptors on the recipient cell surface^70^, enter the recipient cell cytoplasm, or in some cases transfer genetic material and transcription factors to the recipient cell nucleus^26,33,71^. The pathophysiologic consequences of these interactions have been extensively investigated in cancer, in which EV/EP-mediated cell–cell communication can facilitate tumourigenesis and the development of pre-metastatic niches^72,73^. Moreover, the role of EV/EPs in modulating inflammation and immune responses both in cancer^74,75^ and autoimmune diseases^76–78^ has garnered particular interest. However, much of the research in this area to date has focused on EV/EP protein and RNA functionality rather than the role of DNA. In this section, we discuss the functional roles of EV/EP DNA in immune modulation and changes in gene expression (Fig. 3).

### Inflammation and immunity

EV/EP DNA activates a variety of pro-inflammatory signalling pathways in multiple diseases. Exogenous DNA has been shown to bind and activate the cytosolic DNA damage receptor cyclic GMP-AMP synthase (cGAS), followed by subsequent recruitment and activation of stimulator of interferon genes (STING), translocation of transcription factors to the nucleus and expression of pro-inflammatory genes^79^. In cancer, these cytokines recruit and activate CD8+ T cells to promote tumouricidal effects. cGAS-STING signalling was implicated in chemotherapy-treated breast cancer in a study by Kitai et al.^80^, in which DNA-containing S-EVs stimulated cytokine release from dendritic cells (DCs). Abrogation of DC cytokine release in STING knockout models demonstrated the role of cGAS-STING, and by extension S-EV DNA, in modulating the DC inflammatory response. In a subsequent study, Diamond et al.^81^ collected S-EVs from irradiated tumour cells and showed that when taken up by DCs, the radiation-damaged DNA avoided degradation by the cytoplasmic nuclease TREX1. In doing so, the tumour-derived EV DNA was able to activate cGAS-STING and mediate an anti-tumour immune response. This phenomenon is supported by previous studies not related to EV/EPs that also demonstrate the role of irradiated DNA in DC-mediated anti-tumour immunity^82–84^.

EV/EP DNA is also involved in cGAS-STING modulation outside of cancer. In malaria, erythrocytes infected with the malaria parasite release parasitic DNA in EVs that are taken up by monocytes, in which cGAS-STING is activated^85^. This EV-DNA-mediated inflammation likely contributes to innate immune responses and crosstalk with adaptive immunity. When DCs and T cells interact during T-cell priming, the T-cell releases EVs containing both gDNA and mtDNA, which are taken up by the DC^66^. This exogenous DNA activates cGAS-STING signalling and up-regulation of inflammatory genes, conferring upon the DC a more resistant “primed” phenotype against viral infection.

Interestingly, mtDNA has been implicated as a bona fide stimulator of the cGAS/STING axis^10^. In 2016, two groups demonstrated the inflammatory potential of mtDNA in the context of SLE^12,86^. Importantly, oxidation of DNA causes structural changes that impart resistance to degradation by nucleases, thus allowing increased activation of cGAS/STING^12,81,82,86^. Because of its proximity to mitochondrial reactive oxygen species generation, mtDNA tends to undergo oxidation and become more interferogenic. The inflammatory potential of mtDNA can be modulated by other biophysical alterations in addition to oxidative damage. Andreeva et al. demonstrated that mitochondrial transcription factor A (TFAM) imparts a structural conformation to mtDNA that enhances its ability to activate cGAS, suggesting that mtDNA is a more potent activator of cGAS than is gDNA^87^. These findings support earlier work by West et al.^88^, who detailed a similar TFAM-dependent model for mtDNA stimulation of cGAS-STING. While the potential pro-inflammatory interaction of mtDNA and cGAS has yet to be fully realised in the context of EV/EP biology, EV mtDNA has been implicated as an activator of other inflammatory pathways. Tsilloni et al.^89^ proposed EV-derived mtDNA as a key activator of the inflammatory cytokine IL-1 in the brains of children with autism spectrum disorder after finding increased mtDNA levels in these patients. However, patients with greater neural inflammation were also found to have increased total EV levels, and there is not sufficient evidence in this study to definitively implicate EV mtDNA as the primary mediator of IL-1 secretion.

EV DNA-mediated immune modulation can also contribute negatively to patient outcomes in other disease contexts. The chemotherapeutic agent irinotecan was shown to induce large scale release of DNA via EVs from intestinal epithelial cells^90^. Uptake of the EV DNA by nearby innate immune cells resulted in activation of the AIM2 inflammasome and a subsequent inflammatory response resulting in intestinal toxicity. The inflammation induced by EV/EP DNA can have drastically different outcomes depending on disease context. Therefore, further elucidation of these immune-modulatory effects is needed before EV/EP DNA can be appropriately investigated as a vehicle or target for therapy.

### Transfer of genetic material

The uptake of EV/EP DNA into the cytosol can also modulate other endogenous signalling pathways in recipient cells. Seo et al.^91^ generated hepatic cell carcinoma mouse models deficient in alcohol dehydrogenase 2 and found that mice treated with alcohol had increased hepatocyte release of EVs containing mtDNA. This EV mtDNA, when transferred to neighbouring non-cancerous cells, caused up-regulation of several oncogenic pathways. A similar study demonstrated the tumourigenic effects of EV mtDNA in potentiating the radiation-induced bystander effect, in which the phenotype of irradiated cells is transferred to non-irradiated “bystander” cells. Using DNase treatment and mitochondrial depletion assays, EV mtDNA from irradiated cells was shown to play a key role in inflicting chromosomal instability on nearby non-irradiated cells^92^. However, the molecular mechanism by which EV mtDNA induced phenotypic changes in recipient cells was not investigated. Moreover, the lack of EV gDNA in this model seems to contrast with the proposed role of EVs in clearing damaged DNA from cells with genomic instability^50^. An earlier study exploring this difference in functionality between genomic and mitochondrial sources of EV DNA showed that while cancer-cell-derived EVs contained both forms of DNA, only the gDNA was found to localise to the recipient cell nucleus and mediate up-regulation of certain genes^93^. Although the mtDNA did not enter the nucleus, its potential functional role in the recipient cell cytosol was not addressed. In addition, the increase in expression of specific genes could not be directly attributed to transcription of the exogenous EV DNA, suggesting that modulation of gene expression could be triggered by pathways downstream of EV DNA in the cytosol.

Interestingly, several studies have demonstrated that EVs can mediate horizontal gene transfer (HGT) and transcription of exogenous EV DNA in the recipient cell nucleus. In powerful series of experiments, Fischer et al.^60^ transfected bone marrow mesenchymal stromal cells with an exogenous DNA tag and treated non-labelled cells with EVs from labelled cells. Subsequent sequencing revealed the exogenous DNA tag to be stably integrated into the recipient cell genome.

The ability of horizontally transferred genomic content to influence recipient cell phenotypes was shown in the context of coronary artery disease as well. EVs carrying the sex-linked SRY gene transferred their DNA contents to monocytes and endothelial cells, in which expression of the exogenous SRY resulted in up-regulation of adhesion pathways^94^. These findings were supported by clinical data showing that patients with the SRY gene in their plasma EVs experienced higher rates of atherosclerosis, a result of up-regulated adhesion pathways, than did those with EVs that did not contain SRY DNA.

Furthermore, EV-mediated HGT may not be limited to gDNA. In patients with late-stage breast cancer, cancer-associated fibroblasts were shown to package mtDNA into EVs, which, when taken up by hormone therapy-resistant breast cancer cells, contributed to up-regulation of mitochondrial genes necessary for oxidative phosphorylation^95^. This increase in oxidative phosphorylation potential allowed for the cancer cells to escape the therapy-induced dormant state, thus conferring them with resistance to treatment. Transfer of human papillomavirus DNA via serum EVs into triple negative breast cancer stromal cells has also been shown to impart an aggressive and therapy-resistant phenotype on recipient cells^96^.

These results illustrate the potential for tumour cell-derived EV/EP DNA to impart an oncogenic phenotype on nearby non-cancerous cells. Fortunately, these mechanisms also represent promising targets for therapy. A full understanding of the EV/EP DNA-mediated anti-tumour immune response might eventually allow for potentiation of these pathways as novel immunotherapy approaches in cancer. EV/EP DNA can also potentially be used in priming the immune system to defend against infection. Furthermore, blocking transfer of genetic material via EV/EPs may prevent pathophysiologic phenotypes from permeating more cells, with implications in limiting tumour severity and coronary artery disease.

## EV/EP DNA as a disease biomarker

EV biomarkers have demonstrated potential clinical utility in monitoring disease progression and predicting outcomes to treatment in cancer patients^97,98^. MicroRNA (miRNA) has to date been the most thoroughly investigated EV constituent, as many EV populations carry clinically relevant miRNA^97^. Conversely, cfDNA is seen as the definitive molecular biomarker in liquid biopsy^3,99^. As such, cfDNA associated with EV/EPs has recently garnered intense interest as a potential untapped reservoir in DNA-based liquid biopsy. In fact, preliminary studies have suggested that EV DNA could even be superior to plasma cfDNA for certain liquid biopsy applications in cancer^100–102^. In this section, we provide a brief overview of the work being done to evaluate the effectiveness of EV/EP DNA as a molecular biomarker in various diseases.

### Blood-based liquid biopsy

The earliest study proposing the potential role for EV DNA as a cancer biomarker was conducted by Thakur et al.^16^, in which they found BRAF and EGFR mutations in S-EV DNA that reflected the parent cell genotype in pre-clinical models. Shortly thereafter, oncogenic KRAS and TP53 mutations were found in S-EV DNA isolated from the serum of patients with pancreatic cancer^17^. These promising results triggered a landslide of work demonstrating the mutational status of various tumours reflected in their plasma EV-derived DNA. Much of this research has continued to explore plasma EV DNA in pancreatic cancer, with multiple lines of evidence pointing to KRAS and TP53 mutations in EV DNA as bona fide molecular biomarkers for monitoring tumour presence, progression and outcomes^103–107^.

Despite the apparent rapid progress in this field, inconsistent and controversial results have emerged due to unreliable and unstandardised separation methods^108^. Even newer approaches employed in DNA mutation detection, such as microfluidic isolation of plasma EVs, enrich specifically for classically described exosomes instead of diverse EV/EP populations^107^. Garcia-Romero et al. highlight a possible advantage of DNA from multiple EV populations over cfDNA as a biomarker in neurological malignancies. In xenograft mouse models of glioma with an intact blood-brain barrier, tumour-derived L-EVs and S-EVs were found in peripheral blood^109^. Importantly, they further demonstrated that tumour-derived DNA could only cross the blood–brain barrier when associated with EVs, thereby presenting a novel blood–based liquid biopsy approach in cancers typically monitored by less accessible cerebrospinal fluid DNA biomarkers.

### Other body fluids

EV/EP DNA in other fluids represents additional avenues for accessing DNA biomarkers. In lung adenocarcinoma, fluid from malignant pleural effusions harboured EV DNA with EGFR mutations that reflected the tumour genotype more accurately than conventional cytology approaches^110,111^. A subsequent study found a broad array of tumour-derived mutations reflected in EV DNA isolated from pleural effusion supernatant^112^, further reinforcing this approach as a viable liquid biopsy option in lung cancers. The shortfall of this technique, however, is that it requires malignant pleural effusion, which is not present in all lung cancers. To address this issue, Hur et al.^113^ subjected non-small-cell lung cancer patients to bronchioalveolar lavage with subsequent isolation of EV DNA from this fluid. While this EV DNA showed increased specificity and sensitivity as a biomarker when compared with blood-derived cfDNA, bronchioalveolar lavage is relatively invasive, and its effectiveness as a liquid biopsy technique may not outweigh the patient discomfort evoked from this procedure. Far less invasive methods have been demonstrated in diseases of the urinary tract, such as kidney disease^114^ and bladder cancer^115^, in which EV DNA isolated from urine represents a novel biomarker in monitoring disease progression. Although these approaches are limited to diseases in which these biological fluids are relevant, they nonetheless demonstrate EV DNA as a molecular biomarker that can inform clinical decision-making.

## Conclusion

With its seemingly endless potential as a target for therapy and liquid biopsy in a variety of disease states, EV/EP DNA will garner continued interest in the coming years. Therefore, the standardisation of EV/EP DNA isolation and analysis techniques is crucial to ensure our complete understanding of this clinically useful entity. With unreliable and low-resolution EV isolation techniques rapidly becoming outdated, the findings of many of the studies discussed in this review must be validated using newer, more high-resolution approaches. Moreover, differences in EV/EP DNA distribution, localisation and structure must be further investigated, as these characteristics will have major implications with regards to the clinical utility of EV DNA. DNA derived from EVs and EPs is poised to become an increasingly important factor in fields ranging in scope from the anti-cancer immune response to kidney disease screening, with widespread impact on the future of treatment and monitoring of many diseases.
